# Intra-Renal Angiotensin Levels Are Increased in High-Fructose Fed Rats in the Extracorporeal Renal Perfusion Model

**DOI:** 10.3389/fphys.2018.01433

**Published:** 2018-10-10

**Authors:** Rodrigo Yokota, Fernanda Aparecida Ronchi, Fernanda Barrinha Fernandes, Zaira Palomino Jara, Rodolfo Mattar Rosa, Ana Paula de Oliveira Leite, Patricia Fiorino, Vera Farah, Nilberto Robson Falcão do Nascimento, Manassés C. Fonteles, Dulce Elena Casarini

**Affiliations:** ^1^Nephrology Division, Department of Medicine, Federal University of São Paulo, São Paulo, Brazil; ^2^Laboratory of Renal, Cardiovascular, and Metabolic Physiopharmacology, Center for Health and Biological Sciences, Mackenzie Presbyterian University, São Paulo, Brazil; ^3^Superior Institute of Biomedical Sciences, Ceará State University, Fortaleza, Brazil

**Keywords:** renal, fructose, renin angiotensin system, perfused kidney, physiology

## Abstract

Overconsumption of fructose leads to metabolic syndrome as a result of hypertension, insulin resistance, and hyperlipidemia. In this study, the renal function of animals submitted to high fructose intake was analyzed from weaning to adulthood using *in vivo* and *ex vivo* methods, being compared with a normal control group. We investigated in *ex vivo* model of the role of the renin Angiotensin system (RAS) in the kidney. The use of perfused kidney from animals submitted to 8-week fructose treatment showed that high fructose intake caused metabolic and cardiovascular alterations that were consistent with other studies. Moreover, the isolated perfused kidneys obtained from rats under high fructose diet showed a 33% increase in renal perfusion pressure throughout the experimental period due to increased renal vascular resistance and a progressive fall in the glomerular filtration rate, which reached a maximum of 64% decrease. Analysis of RAS peptides in the high fructose group showed a threefold increase in the renal concentrations of angiotensin I (Ang I) and a twofold increase in angiotensin II (Ang II) levels, whereas no change in angiotensin 1-7 (Ang 1-7) was observed when compared with the control animals. We did not detect changes in angiotensin converting enzyme (ACE) activity in renal tissues, but there is a tendency to decrease. These observations suggest that there are alternative ways of producing Ang II in this model. Chymase the enzyme responsible for Ang II formation direct from Ang I was increased in renal tissues in the fructose group, confirming the alternative pathway for the formation of this peptide. Neprilysin (NEP) the Ang 1-7 forming showed a significant decrease in activity in the fructose vs. control group, and a tendency of reduction in ACE2 activity. Thus, these results suggest that the Ang 1-7 vasodilator peptide formation is impaired in this model contributing with the increase of blood pressure. In summary, rats fed high fructose affect renal RAS, which may contribute to several deleterious effects of fructose on the kidneys and consequently an increase in blood pressure.

## Introduction

Metabolic syndrome (MS) is a disease of modern civilization, being strongly correlated with lifestyle (diet and physical activity), and increase has been linked to the intake of high-fructose corn syrup associate a cluster of common pathologies (Zimmet et al., 2001; Ferder et al., 2010; De Angelis et al., 2012; Chou et al., 2017). High consumption of fructose, a sugar widely used in the western world, is associated with pathological changes, including MS, obesity, glucose intolerance, type II diabetes, fatty liver and other liver diseases, hypertension, and cardiovascular diseases, in both experimental and clinical studies (Elliott et al., 2002; Malik et al., 2010a; Morris et al., 2012; Madani et al., 2015).

Fructose fed rats have been used as a model for MS exhibiting several metabolic changes, similar to those observed in the clinical presentation in humans (Rayssiguier et al., 2006; Sanchez-Lozada et al., 2007). In experimental models, high fructose consumption induces hypertension, dyslipidemia, central obesity, body weight increase, insulin resistance, and hepatic steatosis (Suzuki et al., 1997; Farah et al., 2006; Tran et al., 2009a; Mamikutty et al., 2015; Xu et al., 2017). Moreover, we have observed a correlation between parasympathetic impairment and insulin resistance in fructose-fed rats (Van Gaal et al., 2006). Additionally, we have shown a nocturnal hypertension associated with increase in blood pressure variability and brain catecholaminergic activation in this model (Sanchez-Lozada et al., 2007). We have also observed that the time of fructose intake affects the metabolic and cardiovascular parameters in mice (Rayssiguier et al., 2006). More recently, we have shown that fructose-fed animals exhibited no further change in their metabolic parameters when stimulated by NaCl. However, NaCl exacerbated fructose-induced cardiac functional damage, as evaluated by echocardiography and documented in cardiac morphology and diastolic function (Araujo et al., 2016).

The MS is also associated with risk for development of chronic kidney disease (CKD), which is correlated with cardiovascular changes observed in mice (Cunha et al., 2007). The literature has shown a strong correlation between MS and development of microalbuminuria and CKD. Moreover, the risk for developing CKD is progressively increased with the number of criteria that constitutes the MS (Chen et al., 2004), and there was a positive correlation between microalbuminuria and MS features in diabetic American natives (Hoehner et al., 2002).

Studies have confirmed the influence of fructose-rich diet introduced in the early weeks after birth to adulthood (Patel et al., 2009). Recently, we have observed that early exposure to high fructose intake caused metabolic changes, such as hyperglycemia, insulin resistance, and dyslipidemia, associated with hypertension and cardiovascular disautonomia when provided to rats from weaning to adulthood (Araujo et al., 2016; Chou et al., 2017). These modifications are examples of how metabolic programming can modify the outcome of diseases related to metabolic failure in adult life.

There is increasing evidence that excessive intake of fructose may bring several health disturbances, including high blood pressure and MS, causing fatty liver and accelerating renal disease (Elliott et al., 2002; Chen et al., 2004). Sanchez-Lozada et al. (2007) reported that administration of fructose (60% diet) to rats induces renal hypertrophy, with tubular cell proliferation and low-grade tubule interstitial cell injury, generating chemotactic factors such as monocyte chemo-attractant protein-1 by tubular cells and intercellular adhesion molecule-1 in renal microvascular endothelial cells. In addition to these effects, high fructose concentration exacerbates proteinuria, worsens renal function, and accelerates glomerulosclerosis in the remnant kidney model. In the same study, they reported outer cortical glomeruli arteriolar afferent resistance and glomerular hypertension in association with the development of preglomerular vascular disease (Sanchez-Lozada et al., 2007).

The mechanisms by which MS can induce renal diseases are still obscure, but the role of renin angiotensin system (RAS) has been highlighted since experimental studies have shown that fructose overconsumption promotes activation of this endocrine axis (Shinozaki et al., 2004; Bizeau and Pagliassotti, 2005; Farah et al., 2006; Bantle, 2009). Vidotti et al. (2004) described that high glucose levels increase angiotensin II (Ang II) generation in mesangial cells as a result of activation of renin and its substrate angiotensinogen through an intracrine action, mediating the proliferative and inflammatory effects of this peptide (Vidotti et al., 2004). The result of this mechanism can contribute to cell proliferation, matrix expansion, inflammation contributing to the glomerular sclerosis described in diabetic nephropathy (Vidotti et al., 2004).

In fact, our group showed that the nocturnal hypertension induced by high fructose diet in mice could be related to angiotensin system activation (Farah et al., 2006). In addition, we also used the angiotensin AT (1a) *knockout* mice model and found no increase in blood pressure after high fructose diet (Farah et al., 2007). However, further studies are necessary to understand the role of renal RAS in the development of changes observed in MS after as induced by high fructose diet.

Therefore, a deeper understanding of molecular mechanisms originated from chronic fructose consumption, which might cause direct renal effects in rats from weaning to adulthood, and the role of RAS in are necessary in this model of kidney failure.

## Materials and Methods

### Experimental Model

Male Wistar rats (3-week old; approximate weight: 50 g) were observed for 8 weeks. Animals were housed in standard cages (with free access to food and water) and maintained at a constant temperature (23°C) on a 12-h light-dark cycle. All surgical procedures and protocols were in accordance with the Guidelines for Ethical Care of Experimental Animals and were approved by the Research Ethics Committee of the Federal University of Sao Paulo (No. 0375/2010). Animals were randomly divided into two groups of six animals: control group (CG), which received regular water during the investigation period, and fructose group (FG), which received regular water containing fructose (10%) for 8 weeks beginning at weaning. Body weight, daily food intake, and water consumption were monitored during all the experimental period. Rats were transferred to metabolic cages twice/one weeks before surgery and on the 8th week, and collected 24-h urine output.

### Glucose Tolerance Test (GTT) and Area Under the Curve (AUC)

The GTT was performed after the 8-week experimental period and after 8-h fasting. A glucose load (1.5 g per kg of body weight) was injected intraperitoneally as a bolus, and blood glucose levels were determined using a glucometer (Accu-Chek Advantage Roche Diagnostics^®^). The blood samples were taken from a cut made on the tip of the tail were taken at baseline and 15, 30, 60, and 90 min after glucose administration. The AUC was calculated using the GraphPad Prism 5 (GraphPad Inc., San Diego, CA, United States) software.

### Cardiovascular Assessments

At end of the protocol (8th weeks treatments), the animals were anesthetized with a ketamine-xylazine solution (50:10 mg/kg ratio; *ip*) to implant arterial and vein catheters for direct measurements of the arterial pressure (AP) and heart rate (HR), respectively. Rats were studied after 24 h after catheter placement. The animals were conscious and allowed to move freely during the experiment. An arterial cannula was connected to transducer (Hewlett-Packard 1280, United States) coupled to a signal amplifier (General-Purpose Amplifier, Stemtech, Inc.,) and AP measurement was discontinued and conscious animals were recorded for 30 min period using a microcomputer equipped with an analog – to – digital converter board (WinDaq, 4 kHz, DATAQ Instruments). Beat-to-beat values of mean AP (MAP) were determined and the heart rate (HR) was calculated (WinDaq, DATAQ Instruments, Inc., United States).

### Metabolic Measurements

After the cardiovascular procedures, the animals were euthanized and total blood was used to determine hematocrit. The serum was collected and stored at −20°C to evaluate triglycerides, total cholesterol, HDL, and LDL using the chemiluminescent method (Lab Test, Brazil).

### Perfused Rat Kidney Assay

At the end of the 8th week, the rats were anesthetized with sodium pentobarbital (50 mg/kg body weight). After a careful dissection of the right kidney, the right renal artery was cannulated via the mesenteric artery (without interrupting blood flow to avoid ischemic damage) and placed into the perfusion line, thus isolating the kidney from endocrine and neural influence. Then, the animals were euthanized according to Fonteles et al. (1983).

The perfusate consisted of a modified Krebs-Henseleit solution (MKHS) containing: NaCl (118 mM), KCl (1.2 mM), KH_2_PO_4_ (1.18 mM), MgSO_4_.7H_2_O (1.18 mM), CaCl_2_.2H_2_O (2.5 mM), and NaHCO_3_ (25 mM). Bovine serum albumin (6 g%) was added to the MKHS, which was dialyzed (48 h; 4°C) against a volume 10 times larger of regular MKHS. The isolated perfused rat kidney assay was performed according to methods previously described by Bowman (1970), and modified by Fonteles et al. (1983, 1998), by introducing a silastic membrane oxygenator into the perfusion line (P_O2_ = 450–500 Torr). Immediately before the beginning of each perfusion, urea (8.3 mM), inulin (1.25 mM), and glucose (5.55 mM) (Sigma, St. Louis, MO, United States) were added to the solution, and the pH value was adjusted to 7.40 and placed and maintained at 37°C during the experimental period. During experiments, the right kidney was perfused for 30 min as an equilibration period for adjustment of the preparation to the new conditions (perfusion pressure) and blood washout, and after this was perfused for 90 min. The perfusion pressure and flow rate were recorded continuously. However, the flow rate of 20 ml/min was constant during the experiment. Urine and perfusate samples were collected at 10-min intervals for biochemical analysis. The glomerular filtration rate (GFR) and osmolar clearance (C_osm_) were determined using standard clearance formulas as described elsewhere (Martinez-Maldonado and Opava-Stitzer, 1978; Lima et al., 1992; Fonteles et al., 1998). The glomerular filtration rate was determined by the inulin clearance, using the method described by Walser et al. (1955) and modified by Fonteles et al. (1983). Inulin was determined by a colorimetric method after direct acid hydrolysis with acetic acid and hydrochloric acid and using diphenylamine as color reagent. Osmolality of both perfusate and urine were measured using vapor pressure osmometer (Vapro 5520; Wescor, Inc., Logan, UT, United States).

### Protein Quantification

Protein concentration was estimated for left kidney homogenates by the Bradford (1976) method, using Coomassie Blue reagent (1:5; Bio-Rad, Brazil). Absorbance (595 nm) values were read at the Infinite F-200 (Tecan; Grödig, Austria). Calculation was based on a bovine serum albumin standard curve; values were expressed in mg/ml.

### Enzymatic Activities

#### ACE

Left kidney tissues (1 g tissue: 10 ml buffer) were homogenized in borate buffer (400 mM; pH 7.20; 10 mL) containing NaCl (900 mM), sucrose (340 mM), and the inhibitors *p*-hydroxymercuribenzoate (*p*-OHMB; 0.1 mM/mL homogenate) and phenylmethanesulfonyl fluoride (PMSF; 100 mM/mL homogenate). The homogenates were centrifuged (1000 *g*; 10 min) and the supernatants were frozen (−20°C) until analysis. The ACE catalytic activity was determined fluorometrically as described by Casarini et al. (Foster et al., 2003). Briefly, an aliquot (10 μL) of homogenate was incubated with Z-Phe-His-Leu (ZPhe-HL; 1 mM; 200 μL). The enzymatic reaction was stopped by the addition of NaOH (0.28 N; 1.5 mL) and incubated with *o*-phthaldialdehyde (20 mg/mL methanol; 100 μL; 10 min). The fluorescence reaction was stopped by the addition of HCl (3 N; 200 μL). The dipeptide His-Leu (L-HL) thus released was measured fluorometrically (λ:365 nm; λ_em_:495 nm) using the F-200 (Infinite Model; Tecan; Grödig, Austria) fluorometer. The results are expressed as mU/mL for serum and mU/mg protein for tissue homogenate.

#### ACE2

Left kidney samples were homogenized in 50 mM Tris buffer pH 7.5 (1 g tissue: 10 mL buffer) containing proteases inhibitors (*Complete mini EDTA free, Roche*, United States). Homogenates were centrifuged twice (15000 rpm, 15 min, −4°C) and supernatants stored at −20°C. ACE2 activity was determined in spectrofluorimeter (*Tecan*, Switzerland), using the substrate Mca-APK-Dnp (30 μM, excitation 320 nm, emission 420 nm). Buffer (Tris–HCl 50 mM, NaCl 1 M, ZnCl_2_ 10 μM, captopril 10 μM, pH 6.5) and samples (2 μL) were pre-incubated for 30 min in the presence or the absence of ACE2 inhibitor (DX600, 20 μM). Substrate was added and samples were read in 0 and 60 min. Arbitrary units were registered, calculations were done based on a fluorescence standard curve (OMNIMMP) and the time point 0 was used as internal blank. Values were normalized by the protein concentration (nmol/min/mg).

#### Chymase

Chymase activity was determined in spectrofluorimeter (*Tecan*, Switzerland), using the substrate Abz-AIKFFSAQ-EDDnp (10 μM, excitation 320 nm, emission 420 nm). An aliquot of kidney homogenate (10 μl) was incubated in 100 mM Tris–HCl buffer, pH 7.4 at 37°C. The same procedure was performed in the presence of the specific inhibitor chymostatin (100 μM). The enzyme activity was defined as the amount of substrate sensitized by chymostatin and corrected by protein concentration of each sample. The activity was expressed as μM/min/mg protein.

#### NEP

The NEP activity was measured after pre incubation of 10 μl of kidney homogenate at 37°C for 30 min in 50 mM Tris–HCl buffer, pH 7.4, in plates from the Infinite 200 (*Tecan*, Switzerland), followed by Abz(d)R-G-Leu (10 μM, excitation 320 nm, emission 420 nm). The same procedure was performed in the presence of the specific inhibitor Thiorphan (2 μM). The proteolytic activity was expressed as μM/min/mg protein.

### Western Blotting Analysis

Sodium dodecyl sulfate polyacrylamide gel electrophoresis (7.5%) was developed with a protein equivalent (100 μg), which was lyophilized and dissolved in sample buffer (30 μL). The following steps were performed according to the method described by Laemmli (Vidotti et al., 2004). After electrophoresis, proteins were transferred onto a nitrocellulose membrane (HYBOND; GE Healthcare; Piscataway, NJ, United States). The primary (1:250; biotinylated; ACE 9B9; Chemicon, International Inc., Temecula, CA, United States) antibody was incubated overnight. The secondary anti-mouse IgG (1:2000; GE healthcare; Switzerland) antibody was used when necessary. The subsequent steps were performed using the streptavidin-alkaline phosphatase system (Amersham Pharmacia Biotech; Sweden). Bands were revealed with the NBT/BCIP substrates (Bio-Rad; United States). Optical densities were analyzed and quantified using the GS-800 Calibrated Densitometer and QuantityOne (Bio-Rad) software. Protein expression was measured in pixels/mm^2^ and normalized to actin expression.

### Angiotensin Quantification by High-Performance Liquid Chromatography (HPLC)

Angiotensin peptides were quantified by High Performance Liquid Chromatography (HPLC), according to the method previously described by our group (Almeida et al., 2006; Ronchi et al., 2007).

Kidneys (left) were homogenized in sodium phosphate buffer (100 mM; pH 7.2), containing sucrose (240 mM), NaCl (300 mM), and the following protease inhibitors: potassium EDTA (25 mM), *o*-phenanthroline (0.44 mM), pepstatin A (0.12 mM), and 4-chloromercuribenzoic acid (1 mM) in a proportion of 20 mL per gram of tissue. The homogenates were centrifuged (14,000 *g*; 20 min; 4°C). The samples were extracted using C_18_ Sep-Pak columns and analyzed by HPLC. The peptide was separated by HPLC (Shimadzu System; Kyoto, Japan) using an Aquapore reversed-phase column (250 mm × 4.6 mm; ODS 300; 7 μm; PerkinElmer; São Paulo, São Paulo, Brazil), using a linear gradient (5–35%) of the mobile phase B (95% acetonitrile in 0.1% H_3_PO_4_) at a 1.5 mL/min flow rate (40 min) (Rosa et al., 2016). The HPLC column was calibrated under the same conditions using synthetic standards, and the peptides angiotensin I (Ang I), angiotensin II (Ang II), and angiotensin 1-7 (Ang 1-7) were detected with absorbance (l = 214 nm). The results were expressed as pmol per gram of tissue.

### Statistical Analysis

Values were represented as median (X) ± SE. Data from fructose group were compared with control group by two-tail non-parametric *t*-test (Mann-Whitney test). The significance level (*P*-value) of 5% was considered statistically significant.

## Results

**FIGURE 1:**
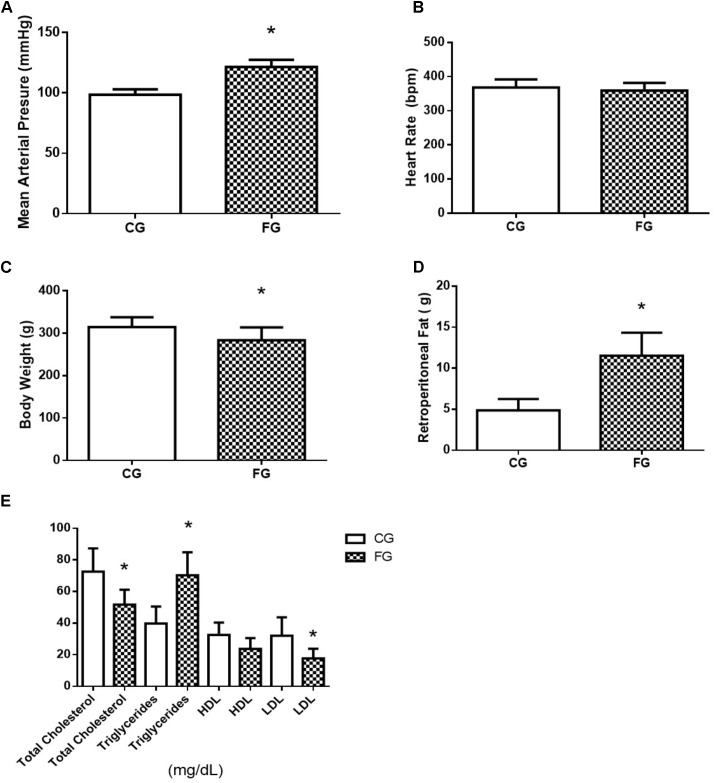
**(A)** Mean arterial pressure; **(B)** heart rate; **(C)** body weight; **(D)** retroperitoneal fat; and **(E)** total cholesterol, triglycerides, HDL, and LDL in control rats (CG) and rats under high fructose diet (FG). The results are expressed as median ± SE (*n* = 6 per group). ^∗^*p* < 0.05 vs. CG.

The high fructose diet induced the following features of MS: glucose intolerance, dyslipidemia, changes fasting blood glucose, and increased blood pressure, but no significant change in the heart rate (**Figures 1A,B**). Although both groups, rats under normal (CG) or high fructose (FG) diets, received the same energy intake and there was no significant change in body weight, the adipose tissue was increased in FG when compared to CG (**Figures 1C,D**). Serum levels of total cholesterol, triglycerides, HDL, and LDL were analyzed at the end of protocol. However, total cholesterol in FG had significantly decreased when compared with CG. In addition, triglycerides in FG showed a significant increase when compared with CG, and LDL in FG showed a significant decrease when compared with the CG, and HDL did not present a significant difference (**Figure 1E**).

**FIGURE 2:**
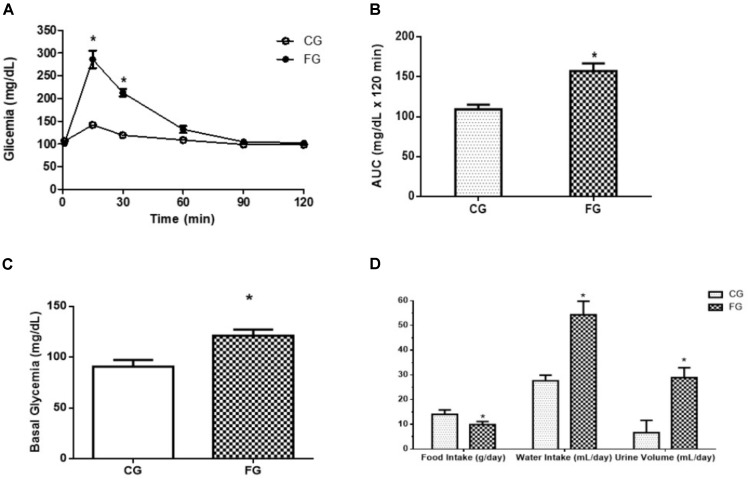
**(A)** Oral glucose tolerance test (OGTT); **(B)** A: glucose concentration (time 0; obtained after 8-h fast); B: area under the curve (AUC) of OGTT; **(C)** fasting blood glucose (basal glycemia); **(D)** food intake, water intake, and urinary volume in control rats (CG) and rats under high fructose diet (FG). The rats were housed individually in metabolic cages (24 h). The results are expressed as median ± SE (*n* = 6 per group). ^∗^*p* < 0.05 vs. CG.

Furthermore, the FG group showed a decreased tolerance to glucose **Figure 2A**, and a 44% increase in the area under the curve in the oral glucose tolerance test **Figure 2B**. The basal glycemic level was increased in the animals that received the high fructose diet **Figure 2C**. At the end of protocol (8th week), the rats were kept in the metabolic cage to evaluate the food and water intake and urinary excretion. The animals in the FG showed less food intake than those in the CG (29% reduction). Interestingly, fructose induced an increase in water intake (97%) as well as urinary excretion (338%) (**Figure 2D**).

**FIGURE 3:**
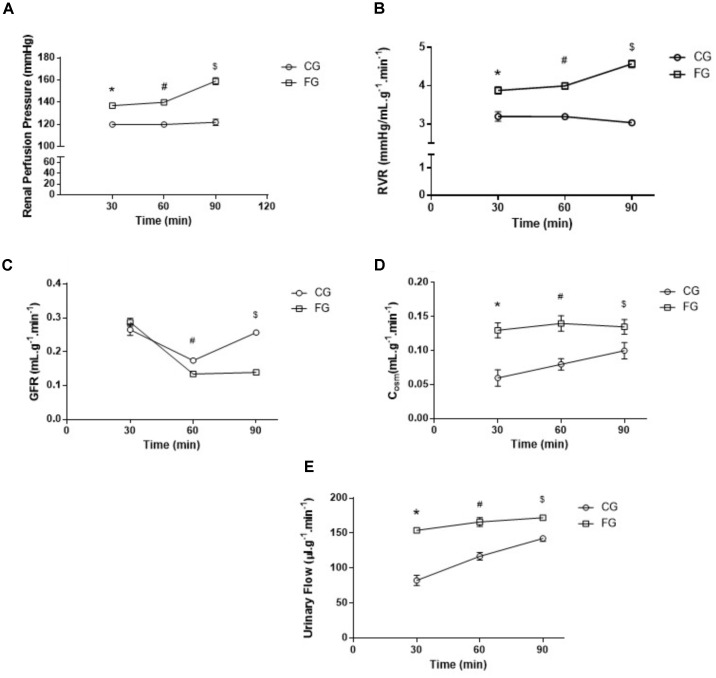
**(A)** Renal perfusion pressure; **(B)** renal vascular resistance (RVR); **(C)** glomerular filtration rate (GFR); **(D)** osmolar clearance (C_osm_); and **(E)** Urinary Flow (UF) of isolated perfused kidneys from control rats (CG) and rats under high fructose diet (FG). The results are expressed as median ± SE (*n* = 6 per group). ^∗^*p* < 0.05 vs. 30 min CG; ^#^*p* < 0.05 vs. 60 min CG; ^$^*p* < 0.05 vs. 90 min CG.

The perfusion pressure in kidneys from rats in a high fructose diet was higher than that observed in kidneys from rats under a normal diet (**Figure 3A**). At the end of the experimental period (90 min), the renal perfusion pressure was determined in the CG (119 ± 3 mmHg) and FG (159 ± 9 mmHg) groups (**Figure 3A**). The reason of this difference was an increased renal vascular resistance in the kidneys from rats under a high fructose diet throughout the experiment (**Figure 3B**).

In addition, the glomerular filtration rate decreased over time in kidneys from rats under a high fructose diet when compared to those under a normal diet. This decrease reached a maximum of 64% in the last experimental period (**Figure 3C**).

As for the osmolar clearance, we observed a significant increase in this parameter throughout the renal perfusion experiment when compared to the controls (**Figure 3D**). In the last experimental period (90 min), the osmolar clearance in the kidneys from rats under a high fructose diet was 81% higher when compared to that in the time-matched controls. The same trend was observed in the urinary flow (**Figure 3E**).

**FIGURE 4:**
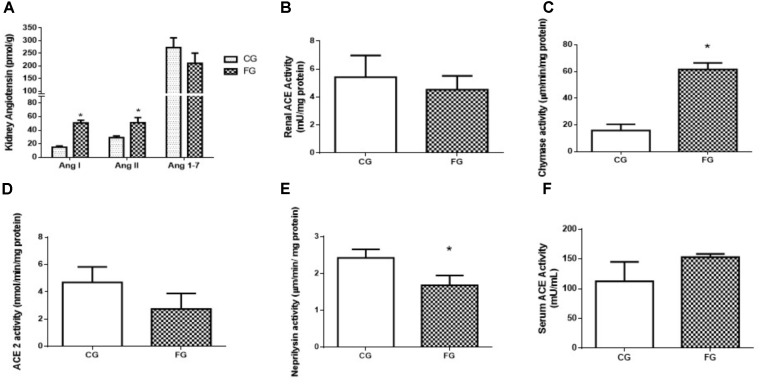
**(A)** Intrarenal levels of angiotensin I, angiotensin II, and angiotensin 1-7. **(B)** Angiotensin converting enzyme (ACE) activity in the kidney, and **(C)** chymase activity in the kidney, **(D)** ACE2 activity in the kidney, **(E)** neprilysin activity in the kidney, **(F)** serum ACE activity from control rats (CG) and rats under high fructose diet (FG). The results are expressed as median ± SE (*n* = 6 per group). ^∗^*p* < 0.05 vs. CG.

Strikingly, an increase was observed in the values for the Ang I (threefold) and Ang II (twofold) concentrations in the kidney from the fructose-fed group as compared to the values obtained in control rats. On the other hand, no significant difference was observed in the values for renal Ang 1-7 concentration between groups (**Figure 4A**). Although we found no significant difference in the renal ACE activity of fructose treated rats (4.26 ± 0.22 vs. 5.89 ± 0.59 mU/mg protein) the tendency of it decrease can be visualized in **Figure 4B**. Chymase activity increased in fructose treated rats (14.9 ± 2.3 vs. 63.5 ± 2.2 μm/min/mg protein) presented in **Figure 4C** and ACE2 activity showed no significant difference (**Figure 4D**). The activity of NEP showed a significant reduction in the fructose group when compared to control (2.43 ± 0.04 vs. 1.72 ± 0.13 μm/min/mg protein) (**Figure 4E**). ACE activity in the serum was increased in these animals (153 ± 3 mU/mL) compared to control rats (103 ± 14 mU/mL; **Figure 4F**), suggesting a dissociation of local and circulating RAS.

**FIGURE 5:**
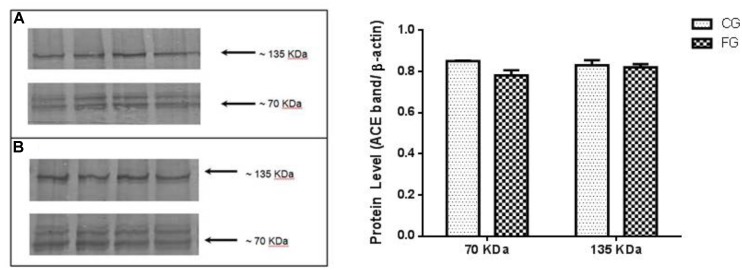
Kidney expression of ACE (135 kDa), similar to the somatic and N-domain ACE (70 kDa) in control rats (CG) and rats under high fructose diet (FG). The results are expressed as median ± SE (*n* = 6 per group). ^∗^*p* < 0.05 vs. CG.

In addition, we could not detect any difference between groups in the protein expression of renal ACE isoforms of both renal (70 kDa; N-domain) and somatic (135 kDa) ACE (**Figure 5**).

## Discussion

In the present study, we evaluated renal function and the role of the kidney RAS in animals submitted to high fructose intake from weaning to adulthood by using *in vivo* and *ex vivo* methods in this model. Our results showed that high fructose intake determined metabolic and cardiovascular changes, which were consistent with those observed in others studies (Farah et al., 2006, 2007; Araujo et al., 2016; Chou et al., 2017; Xu et al., 2017).

Remarkably, isolated perfused kidneys from rats under high fructose diet showed increased renal perfusion pressure throughout the whole experimental period due to increased renal vascular resistance.

Initial analysis of the RAS system in our model showed an increase in ACE activity in serum, but no difference in the ACE activity in kidneys from animals treated with high fructose diet. Remarkably, we observed a significant increase in the renal concentrations of both Ang I and Ang II, but no change in those of Ang 1-7. Therefore, these increased angiotensin levels might contribute to the increased renal vascular resistance in the *ex vivo* perfused kidney.

The present data showed no difference in the body weight of fructose-fed animals. However, an increase was observed in their visceral fat. These data are consistent with those of other studies using this model in rodents (Farah et al., 2007; De Angelis et al., 2012; Chou et al., 2017; Xu et al., 2017). High fructose diet also induced glucose intolerance and high fasting blood glucose. These data are also in agreement to those of previous studies, which suggest that fructose is associated with development of type-2 diabetes and MS (Malik et al., 2010b; Wang et al., 2015). In addition, our data showed that fructose diet induced hypertriglyceridemia, reinforcing the idea that fructose is a lipogenic sugar (Basciano et al., 2005; De Angelis et al., 2009).

Evaluation of arterial pressure from beat to beat in awake animals confirmed an increase in arterial blood pressure, with no change in the heart rate of the fructose group rats. These data are in agreement to those of other studies that showed an association between hypertension and high fructose consumption (Farah et al., 2006; Araujo et al., 2016; Xu et al., 2017). The mechanisms involved in the genesis of fructose-induced hypertension are not clear. Several hypotheses have been proposed, including participation of increased sympathetic tonus and angiotensin system activity (De Angelis et al., 2009, 2012). There are much evidence showing a direct association between excess dietary fructose and activation of sympathetic nervous system. Increased depressor response to alpha-1 adrenergic blockade was demonstrated in mice submitted to high fructose diet, suggesting activation of the sympathetic nervous system induced by fructose intake (Farah et al., 2006). In addition, to this, in rats, pharmacological reduction in sympathetic outflow prevents high fructose diet-induced hypertension in rats (Rosen et al., 1997; Penicaud et al., 1998; Klein and Kiat, 2015). Furthermore, it is believed that excess fructose diet-induced hyperinsulinemia and insulin resistance induce sympathetic nervous system activation (Tran et al., 2009b). Therefore, sympathetic system overactivation could exacerbate high fructose diet-induced metabolic changes.

Evaluation of renal function in rats fed a high fructose diet in metabolic cages showed an increase in water consumption and a consequent increase in urinary volume. These data suggest that fructose does not influence water balance *in vivo*.

In control rats, a stable perfusion pressure was observed throughout the perfusion time. Regarding the group of kidneys from rats in the fructose group, we observed a slight, but significant increase in the perfusion pressure, especially at 90 min of experimentation. It is possible that part of this observation results from angiotensin II production (Rattigan et al., 1995; Fisher et al., 2008). In the present study, we have indeed observed an increase in the renal angiotensin I and II concentrations.

Although the glomerular filtration rate in control rats was constant, in high fructose fed rats we observed a graded decrease that could result from angiotensin II-induced vasoconstriction by activation of AT_1_ receptors in the afferent arteriole (Huang, 1991; Arendshorst et al., 1999; van Rodijnen et al., 2002). In addition, angiotensin II also enhances the tubule glomerular feedback by activation of luminal AT_1_ receptors on the macula densa, leading to afferent arteriole vasoconstriction (Wang et al., 2001). The counter regulation of that by AT_2_ receptor activation is probably blunted since in this model of high fructose-induced renal damage, endothelial dysfunction is present and nitric oxide availability is decreased (Kamata and Yamashita, 1999; Nishimoto et al., 2002). These findings are consistent with those of Sánchez-Lozada and coworkers, who showed that fructose-induced MS results in renal hypertrophy, afferent arteriolopathy, glomerular hypertension, and adrenal vasoconstriction (Sanchez-Lozada et al., 2007).

On the other hand, the osmolar clearance was increased in the kidneys from rats under high fructose diet. This is probably due to an increased pressure diuresis, since renal perfusion pressure was increased in this group with a consequent increase in the delivery of filtrate/fluid to the distal nephron. This diuresis depends on the pressure transmission through the medullary circulation, with increased renal interstitial hydrostatic pressure. Angiotensin II decreases cortical blood flow, but does not affect medullary blood flow. In conditions of high renal perfusion pressure, medullar blood flow increases and, hence, the osmolar clearance and dieresis also increase (Nascimento et al., 2011).

Participation of the RAS in the insulin resistance such as observed in MS is a well-known phenomenon (Putnam et al., 2012). In the present study, we did not observed alterations in renal ACE activity, but a tendency of decrease, and a increase in serum ACE of FG animals. Our results point out to a participation of the RAS in the genesis of fructose-induced hypertension, in accordance with recent literature (Farah et al., 2007; Araujo et al., 2016). For example, increased serum Ang II levels were observed in high fructose-fed rats (Tran et al., 2009b) and increased AT_1_ receptor mRNA levels (Giacchetti et al., 2000).

Our results showed that there was no alteration of ACE activity, but increased chymase activity in FG in renal tissues, that associated with elevated Ang II concentrations suggest that alternative pathways were activated to produce this peptide, as has been shown to occur in the kidney under toxemia as described by Rosa et al. (2016). We can also confirmed that the enzymes tonin, cathepsin, and chymase are involved in this increase in Ang II levels (Rosa et al., 2016). In our high fructose model of kidney damage, we believe that these enzymes may also be responsible for the high Ang II levels. The increase in the Ang I levels detected in the present study also suggest that renin is strongly activated in this model, similar to the results reported by Vidotti et al. (2004) for mesangial cells submitted to high glucose levels (Vidotti et al., 2004; Westwood and Chappell, 2012).

In conclusion, we showed a clear effect of high fructose consumption in the renal angiotensin system. This effect suggests that modulation of enzymes in this system was affected thus causing changes in the production of Ang I and Ang II, which are reflected in the high levels of blood pressure.

## Author Contributions

RY participated in research design, writing of the paper, performance of the research, and data analysis. FR, FF, RR, and ZJ participated in performance of research and data analysis. AL, PF, and NdN participated in performance of research. VF and MF participated in performance of research and writing of the paper. DC participated in performance of research design and writing of the paper.

## Conflict of Interest Statement

The authors declare that the research was conducted in the absence of any commercial or financial relationships that could be construed as a potential conflict of interest.
